# Strategies to Improve Antimicrobial Utilization with a Special Focus on Developing Countries

**DOI:** 10.3390/life11060528

**Published:** 2021-06-07

**Authors:** Brian Godman, Abiodun Egwuenu, Mainul Haque, Oliver Ombeva Malande, Natalie Schellack, Santosh Kumar, Zikria Saleem, Jacqueline Sneddon, Iris Hoxha, Salequl Islam, Julius Mwita, Renata Cristina Rezende Macedo do Nascimento, Isabella Piassi Dias Godói, Loveline Lum Niba, Adefolarin A. Amu, Joseph Acolatse, Robert Incoom, Israel Abebrese Sefah, Sylvia Opanga, Amanj Kurdi, Ibrahim Chikowe, Felix Khuluza, Dan Kibuule, Olayinka O. Ogunleye, Adesola Olalekan, Vanda Markovic-Pekovic, Johanna C. Meyer, Abubakr Alfadl, Thuy Nguyen Thi Phuong, Aubrey C. Kalungia, Stephen Campbell, Alice Pisana, Janney Wale, R. Andrew Seaton

**Affiliations:** 1 Strathclyde Institute of Pharmacy and Biomedical Sciences, University of Strathclyde, Glasgow G4 0RE, UK; amanj.baker@strath.ac.uk; 2 Division of Public Health Pharmacy and Management, School of Pharmacy, Sefako Makgatho Health Sciences University, Pretoria 0204, South Africa; hannelie.meyer@smu.com; 3 School of Pharmaceutical Sciences, Universiti Sains Malaysia (USM), Penang 11800, Malaysia; 4 AMR Programme Manager, Nigeria Centre for Disease Control (NCDC), Ebitu Ukiwe Street, Jabi, Abuja 240102, Nigeria; abiodun.egwuenu@ncdc.gov.ng; 5 Unit of Pharmacology, Faculty of Medicine and Defence Health, Universiti Pertahanan Nasional Malaysia (National Defence University of Malaysia), Kem Perdana Sungai Besi, Kuala Lumpur 57000, Malaysia; runurono@gmail.com; 6 Department of Child Health and Paediatrics, Egerton University, Nakuru, P.O. Box 536, Egerton 20115, Kenya; ombevaom@gmail.com; 7 East Africa Centre for Vaccines and Immunization (ECAVI), Namela House, Naguru, Kampala P.O. Box 3040, Uganda; 8 Faculty of Health Sciences, Basic Medical Sciences Building, University of Pretoria, Prinshof 349-Jr, Pretoria 0084, South Africa; natalie.schellack@up.ac.za; 9 Department of Periodontology and Implantology, Karnavati University, Gandhinagar 382422, India; drsantoshkumar2004@gmail.com; 10 Department of Pharmacy Practice, Faculty of Pharmacy, The University of Lahore, Lahore 54000, Pakistan; xikria@gmail.com; 11 Healthcare Improvement Scotland, Delta House, 50 West Nile Street, Glasgow G1 2NP, UK; jacqueline.sneddon@nhs.scot (J.S.); Andrew.Seaton@ggc.scot.nhs.uk (R.A.S.); 12 Department of Pharmacy, Faculty of Medicine, University of Medicine Tirana, 1005 Tirana, Albania; iris.hoxha@umed.edu.al; 13 Department of Microbiology, Jahangirnagar University, Savar, Dhaka 1342, Bangladesh; salequl@juniv.edu; 14 Department of Internal Medicine, Faculty of Medicine, University of Botswana, Private Bag 0022, Gaborone, Botswana; mwitajc@ub.ac.bw; 15 Department of Pharmacy, Postgraduate Program in Pharmaceutical Sciences (CiPharma), School of Pharmacy, Federal University of Ouro Preto, Ouro Preto 35400-000, Minas Gerais, Brazil; renata.nascimento@ufop.edu.br; 16 Institute of Health and Biological Studies, Universidade Federal do Sul e Sudeste do Pará, Avenida dos Ipês, s/n, Cidade Universitária, Cidade Jardim, Marabá 68500-00, Pará, Brazil; isabellapiassi@gmail.com; 17 Center for Research in Management, Society and Epidemiology, Universidade do Estado de Minas Gerais, Belo Horizonte 31270-901, MT, Brazil; 18 Effective Basic Services (eBASE) Africa, Ndamukong Street, Bamenda P.O Box 5175, Cameroon; lumnyanga@gmail.com; 19 Department of Public Health, University of Bamenda, Bambili P.O. Box 39, Cameroon; 20 Pharmacy Department, Eswatini Medical Christian University, P.O. Box A624, Swazi Plaza, Mbabane H101, Eswatini; folarinamu@gmail.com; 21 Pharmacy Directorate, Cape Coast Teaching Hospital (CCTH), Cape Coast, Ghana; joezy_35@yahoo.com (J.A.); robertincoom30@yahoo.com (R.I.); 22 Pharmacy Department, Keta Municipal Hospital, Ghana Health Service, Keta-Dzelukope, Ghana; isefah1980@gmail.com; 23 Pharmacy Practice Department of Pharmacy Practice, School of Pharmacy, University of Health and Allied Sciences, Ho, Volta Region, Ghana; 24 Department of Pharmaceutics and Pharmacy Practice, School of Pharmacy, University of Nairobi, Nairobi P.O. Box 30197-00100, Kenya; sopanga@uonbi.ac.ke; 25 Department of Pharmacology, College of Pharmacy, Hawler Medical University, Erbil 44001, Iraq; 26 Pharmacy Department, College of Medicine, Chichiri 30096, Blantyre 3, Malawi; ichikowe@medcol.mw (I.C.); fkhuluza@medcol.mw (F.K.); 27 Department of Pharmacy Practice and Policy, Faculty of Health Sciences, University of Namibia, Windhoek 13301, Namibia; dkibuule@unam.na; 28 Department of Pharmacology, Therapeutics and Toxicology, Lagos State University College of Medicine, Ikeja, Lagos 100271, Nigeria; yinkabode@yahoo.com; 29 Department of Medicine, Lagos State University Teaching Hospital, Ikeja, Lagos 100271, Nigeria; 30 Department of Medical Laboratory Science, University of Lagos, Idiaraba, Lagos 100271, Nigeria; olayinka.ogunleye@lasucom.edu.ng; 31 Centre for Genomics of Non-Communicable Diseases and Personalized Healthcare (CGNPH), University of Lagos, Akoka, Lagos 100271, Nigeria; 32 Faculty of Medicine, Department of Social Pharmacy, University of Banja Luka, 78000 Banja Luka, Bosnia and Herzegovina; vanda.markovic-pekovic@med.unibl.org; 33 National Medicines and Poisons Board, Federal Ministry of Health, Khartoum 11111, Sudan; abubakr13@yahoo.com; 34 Department of Pharmacy Practice, Unaizah College of Pharmacy, Qassim University, Unaizah 56264, Qassim 56453, Saudi Arabia; 35 Pharmaceutical Administration & PharmacoEconomics, Hanoi University of Pharmacy, 13-15 Le Thanh Tong, Hoan Kiem District, Hanoi, Vietnam; thuy_ntp@hup.edu.vn; 36 Department of Pharmacy, School of Health Sciences, University of Zambia, P.O. Box 32379, Lusaka 10101, Zambia; chichokalungia@gmail.com; 37 Centre for Primary Care and Health Services Research, School of Health Sciences, University of Manchester, Manchester M13 9PL, UK; stephen.campbell@manchester.ac.uk; 38 NIHR Greater Manchester Patient Safety Translational Research Centre, School of Health Sciences, University of Manchester, Manchester M13 9PL, UK; 39 Department of Global Public Health, Karolinska Institutet, 17177 Stockholm, Sweden; alice.pisana@stud.ki.se; 40 Independent Researcher, 11a Lydia Street, Brunswick, VIC 3056, Australia; socrates111@bigpond.com; 41 Infectious Disease Department, Queen Elizabeth University Hospital, Govan Road, Glasgow G51 4TF, UK; 42 Department of Medicine, University of Glasgow, Glasgow G12 8QQ, UK

**Keywords:** antimicrobials, antimicrobial stewardship programs, antimicrobial resistance, healthcare-associated infections, COVID-19, lower- and middle-income countries, misinformation, patient initiatives, surgical site infections, vaccines

## Abstract

Antimicrobial resistance (AMR) is a high priority across countries as it increases morbidity, mortality and costs. Concerns with AMR have resulted in multiple initiatives internationally, nationally and regionally to enhance appropriate antibiotic utilization across sectors to reduce AMR, with the overuse of antibiotics exacerbated by the COVID-19 pandemic. Effectively tackling AMR is crucial for all countries. Principally a narrative review of ongoing activities across sectors was undertaken to improve antimicrobial use and address issues with vaccines including COVID-19. Point prevalence surveys have been successful in hospitals to identify areas for quality improvement programs, principally centering on antimicrobial stewardship programs. These include reducing prolonged antibiotic use to prevent surgical site infections. Multiple activities centering on education have been successful in reducing inappropriate prescribing and dispensing of antimicrobials in ambulatory care for essentially viral infections such as acute respiratory infections. It is imperative to develop new quality indicators for ambulatory care given current concerns, and instigate programs with clear public health messaging to reduce misinformation, essential for pandemics. Regular access to effective treatments is needed to reduce resistance to treatments for HIV, malaria and tuberculosis. Key stakeholder groups can instigate multiple initiatives to reduce AMR. These need to be followed up.

## 1. Introduction

Antimicrobial resistance (AMR) is a high-priority area across all countries, increasing morbidity, mortality and costs [1,2,3,4,5,6,7], with several studies documenting the clinical and economic impact of AMR [2,3,8,9,10,11,12,13,14,15]. There is considerable resistance to commonly prescribed antibiotics among low- and middle-income countries (LMICs) including Bangladesh, with resistance rates continuing to rise across countries [16,17,18]. Rising AMR rates need to be urgently addressed, with the World Bank (2017) believing that even in a low-AMR scenario, the loss on world output due to AMR could exceed US$1 trillion annually after 2030, and potentially up to US$3.4 trillion annually, unless addressed, equivalent to 3.8% of annual Gross Domestic Product (GDP) [19]—with the costs of AMR appreciably exceeding those of the antibiotics prescribed [13].

Rising AMR rates are due to a number of factors including rising antimicrobial utilization especially in LMICs [20,21], with the overuse of antimicrobials linked with increasing AMR [19,22,23]. Alongside this, the availability of substandard, spurious or falsified antibiotics, high rates of self-medication especially in LMICs often for self-limiting diseases, the extensive use of antimicrobials to prevent and treat diseases in animals considering that their manure is often used in food production and for aquaculture, water pollution, lack of hand hygiene and proper sanitation as well as travelling between countries enhances AMR rates [11,19,21,23,24,25,26,27,28,29,30,31,32,33,34]. In their recent paper, Van Boeckel et al. (2019) found nearly a 3-fold increase in bacteria showing resistance above 50% to antimicrobials in chickens and pigs in LMICs between 2000 and 2018 [35], with resistance rates expected to rise unless addressed.

Low vaccination rates against disease have also enhanced antimicrobial use and AMR, with current concerns that vaccination rates are being further compromised by the COVID-19 pandemic, with its resultant impact on patient movement and available facilities as a result of lockdown and on other measures [11,21,36,37,38,39,40,41,42], exacerbated by vaccine hesitancy generally alongside misinformation [43,44]. We are though seeing approaches to combat this including mobile vaccination clinics [45].

The general overuse of antimicrobials has been worsened by the COVID-19 pandemic [3,46,47,48], with concerns with differential diagnosis between infections such as tuberculosis (TB) and bacterial upper respiratory tract infections (URTIs) from influenza, coughs, fever and COVID-19 [49,50]. Published studies suggest that over 70% of patients with COVID-19 received antibiotics, including broad-spectrum antibiotics, even when not clinically indicated [41,51,52]. Typically, less than 10% of COVID-19 patients appear to have bacterial or fungal infections [53], with some studies suggesting that only 3.2% of COVID-19 infections are co-infections warranting antibiotics [21,54]. In their recent meta-analysis, Langford et al. (2021) found that overall three-quarters of patients with COVID-19 were prescribed antibiotics, which is significantly higher than the estimated prevalence of bacterial co-infections [21,55]. Consequently, the authors concluded that unnecessary antibiotic use is high in patients with COVID-19 [55], which will enhance AMR rates.

Concerns with rising AMR rates and the consequences have resulted in international, regional, national, and local strategies to try and reduce the rises [12,56,57,58,59,60,61,62,63,64,65,66,67,68]. National initiatives include National Action Plans (NAPs) under the direction of the WHO, building on its Global Action Plan [59,60,69,70,71,72,73,74]. We are already seeing countries assess their progress of NAPs against agreed targets, and this will continue [63,75,76].

Alongside this, there are concerns with multi-resistant microorganisms and the implications for patients with TB, human immunodeficiency virus (HIV), malaria, and typhoid, as well as those with co-infections [77]. Early treatment for patients with co-infections with HIV and drug-resistant TB is essential to reduce the significant risk of death [77,78]. There is also ongoing research to better predict patients with MDR-TB and improve future care [79]. Treatment centers, especially in rural areas, are important to monitor treatment progress and provide access to medicines, to enhance successful management and reduce future cases [80].

Overall, we are aware that coordinated activities among all key stakeholders can address over-utilization of antibiotics. Multiple demand-side measures including education of all key stakeholders, prescribing restrictions, and follow-up in pharmacies stabilized or reduced the utilization of antibiotics in former Soviet Union republics including Azerbaijan, the Republic of Srpska, Bosnia and Herzegovina, and Slovenia versus Poland that failed to instigate such measures [61,62,81,82]. In Poland, there was a small but statistically significant average annual increase in total antibiotic consumption between 2007 and 2016 [82].

We are also aware that there are concerns with high prevalence rates of hospital-associated infections (HAIs) in a number of LMICs, with major challenges surrounding the appropriate use of antibiotics to reduce surgical site infections (SSIs) [83,84,85]. The instigation of ASPs in hospitals, including undertaking point prevalence surveys (PPS) to identify areas for quality improvement programs, can improve future antibiotic use and reduce AMR [3,86,87,88,89,90,91,92,93,94,95,96]. There are though manpower and resource challenges with implementing ASPs in hospitals in LMICs [97]. Having said this, successful ASPs have been introduced in hospitals in LMICs to improve antimicrobial prescribing providing guidance to others [3,76,87,98]. Successful programs have also been introduced in ambulatory care in LMICs to improve prescribing and dispensing of antibiotics for essentially viral infections such as acute respiratory infections (ARIs) and including URTIs [64,99,100]. We are also seeing new technologies being developed, including Apps, to provide prescribing advice and other information to physicians and patients to reduce inappropriate use of antibiotics [101]. However, there are concerns with variable adherence to guidelines, possibly exacerbated by guidelines not always considering local resistance patterns, as well as other activities such as community engagement to improve patient care in practice [64,102,103].

We would like to build on these issues and challenges to discuss potential ways to decrease future AMR rates among LMICs. We have chosen LMICs as they currently have the greatest increase in antimicrobial use, the highest rate of self-purchasing of antibiotics, as well as the highest rate of antimicrobial use in hospitals [20,64,104,105]. In addition, mortality rates from AMR are likely to be highest among LMICs, including those in Africa and Asia, by 2050 unless addressed [11,25]. Effectively tackling AMR is crucial for the future of all countries, exacerbated by few new antibiotics currently being developed; however, this is counterbalanced to some extent by developments in vaccine technology [10,19,32,106]. We hope our review paper will consolidate current knowledge in key areas to provide guidance on potential ways forward among all key stakeholders across LMICs to reduce AMR rates, especially where there are continued concerns.

## 2. Materials and Methods

We undertook a narrative review of published papers to document potential ways to enhance appropriate prescribing and dispensing of antimicrobials. We purposely did not undertake a systematic review as an appreciable number of these have already been conducted on different aspects to improve future prescribing and dispensing of antimicrobials [85,86,92,100,104,105,107,108,109,110,111,112,113,114,115,116,117,118,119,120,121,122,123,124,125,126,127,128,129,130,131,132,133,134]. In addition, we did not document the number of publications by country and sector as we are aware that some countries are more prolific in their publications than others, and such analyses may bias the actual situation. We did though divide initiatives up into those directed at hospitals compared with those in ambulatory care. We are aware that issues such as self-purchasing of antibiotics are especially important in LMICs as up to 100% of pharmacies in these countries dispense antibiotics without a prescription despite legislation which can account for up to 93% to 100% of all dispensed antibiotics [123,124,129,135,136]. This because our principal aim was to provide possible future guidance to all key stakeholder groups within LMICs for potential debate based on the considerable experiences of the co-authors from multiple LMICs. We have successfully used this approach before to stimulate debate in other priority healthcare areas to provide future guidance [64,85,137,138,139,140,141,142,143,144,145,146,147].

The World Bank classification was used to break countries down into LMICs or upper income countries where applicable [148]. Similar to previous studies conducted by the co-authors, upper-income countries will only be commented upon where pertinent [64,84,146]. This is because we are aware that there are different practices and beliefs, as well as a greater availability of healthcare professionals and resources, in higher- versus middle- and low-income countries. In addition, in LMICs, the costs of medicines can account for up to 60% or more of total healthcare expenditure, much of which will be out of pocket, with potentially catastrophic consequences for families when members become ill [149,150,151,152,153,154].

There can also be long waiting times for patients to see physicians in primary healthcare centers (PHCs) in LMICs, long distances to travel to PHCs, and pharmacists are typically open later in the day, resulting in patients often seeking their advice as alternatives to physicians [155,156,157,158,159,160]. This is helped by the fact that community pharmacists are typically trusted by patients and less expensive than physicians, with patients not having to pay physician fees when the need arises [99,135,157,161]. However, we are aware that there can be concerns with the knowledge of pharmacists and their assistants regarding antibiotics and AMR [159,162,163,164].

When documenting interventions that have been undertaken to enhance appropriate prescribing and dispensing of antimicrobials, we will break these down into the 4Es to enhance understanding and comparisons, namely Education, Engineering, Economics and Enforcement [165,166]. Education includes developing guidelines or formularies, with adherence to well-constructed guidelines increasingly seen as demonstrating good quality care [147,167,168,169,170,171,172]. This also includes educational and monitoring activities surrounding the WHO AWaRe list of antibiotics [173,174,175]. Instigating Drug and Therapeutic Committees (DTCs) and ASPs in hospitals to provide educational activities to enhance the rational use of medicines including antimicrobials [97,176,177,178,179] is also classified as education. Engineering includes organizational or managerial interventions which incorporate prescribing and quality targets. These include the percentage of antibiotics prescribed according to agreed guidance and the WHO AWaRe list, the percentage of patients not prescribed prolonged antibiotics to prevent SSIs as well as the percentage of patients reaching agreed target blood pressure levels [147,166,169,175,180,181]. Economics includes financial incentives to physicians, pharmacists, patients or hospitals, for instance providing financial incentives to hospitals to improve their patient safety records and/or reduce payments where there are concerns, incentivizing physicians for attaining agreed prescribing targets including adherence to guidelines, or fining pharmacists for illegally dispensing an antibiotic without a prescription [9,147,166,182,183]. Enforcement includes regulations by law such as banning the dispensing of antibiotics in pharmacies without a prescription, compulsory generic substitution as well as national policies outlining the need for DTCs in hospitals and how their activities should be monitored [100,166,177].

No ethical approval was required for this review as we were not dealing with patients. This is in line with previous similar papers published by the co-authors [64,84,105,138,146,147,184,185].

## 3. Results

There are a number of key issues that need to be addressed to enhance appropriate antimicrobial use in patients and reduce AMR. We will break our findings down into key areas within hospitals and subsequently ambulatory care before concluding with guidance on potential activities that can be undertaken among all key stakeholder groups to reduce AMR.

Activities that can be undertaken in hospitals include PPS studies to gain baseline data of current utilization patterns to guide future quality improvement programs as part of ASPs and other activities. Initiatives in ambulatory care center around activities to improve prescribing and dispensing of antimicrobials especially for self-limiting infections such as viral URTIs. These are especially important since, as mentioned, we are aware that up to 100% of pharmacies in LMICs dispense antibiotics without a prescription despite legislation, and this can account for up to 93% to 100% of all dispensed antibiotics [123,124,129,135,136]. Having said this, HAIs are considered as the most adverse event in healthcare delivery [104,186].

### 3.1. Antimicrobial Use in Hospitals

The first consideration within hospitals is to ensure that activities to improve antimicrobial prescribing are given a high priority if this is not already the case. This includes initiatives such as instigating active infection, prevention and control committees (IPC committees) [187,188,189,190,191,192], which can be part of antimicrobial stewardship groups [76,86]. The latter can be part of IPC committees or attached to Drug and Therapeutic Committees (DTCs). In addition, PPS studies can be undertaken and ASPs indicated to improve future antimicrobial use in hospitals (Table 1).

However, there can be issues with the instigation of IPC committees and their effectiveness in hospitals (Table 1), concerns about the knowledge of ASPs among clinicians in LMICs, concerns with resource issues with establishing active ASPs including manpower, as well as a lack of institutional policies in LMICs [97,188,214,215,216]. These issues can be exacerbated by a lack of clear commitment nationally despite the instigation of NAPs, which needs to be addressed to improve future antimicrobial use [213,216].

**Figure 1:**
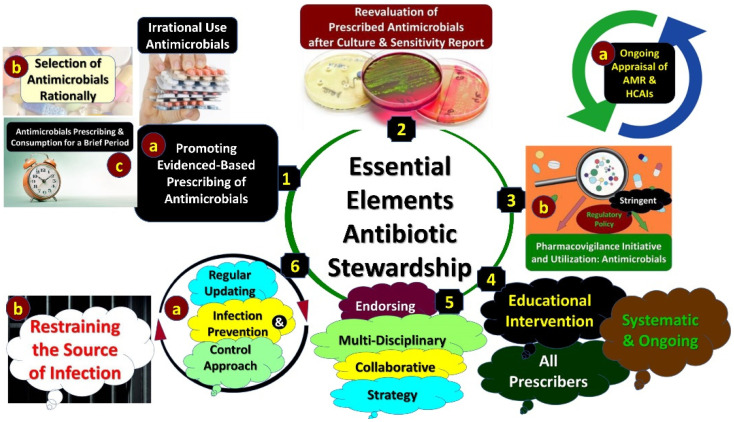
Core elements of any antimicrobial stewardship program.

Figure 1 incorporates the core elements of any ASP, with Kakkar et al. (2020) discussing the potential composition of personnel within antimicrobial stewardship groups in LMICs including physicians, pharmacists, microbiologists, infection control personnel as well as hospital administrators [213]. Ideally, there should be a single focal point, with leaders of ASPs typically being either physicians or pharmacists, with pharmacists increasingly helping to drive forward ASPs in LMICs [87,98,171,211,217]. ASPs can also be part of DTC activities if needed in some hospitals [211].

Box 1 contains a number of indicators that have been used to assess the quality of antimicrobial prescribing within hospitals.

Box 1 **Possible indicators that have been used in LMICs to assess the quality of antimicrobial prescribing (adapted from [88,170,171,193,211,218,219,220]).**

- Availability, ready access and subsequent compliance to local or regional treatment guidelines for key infections. This includes the availability of antibiograms within hospitals to guide empiric use whilst waiting for culture and sensitivity test (CST) findings as well as a potential list of restricted antibiotics depending on physician experience
- Whether antibiotics prescribed are included in the hospital formulary/national essential medicine lists
- Rationale (indication) for antimicrobial use documented in the patient’s notes
- Whether therapy was empiric or targeted based on CST findings
- Documentation of the dose in the patient’s notes
- Documentation of a stop/review date for given antibiotics in the patient’s notes. This can include the rate of de-escalation to a narrower-spectrum antibiotic where appropriate
- Documentation of any missed doses of antibiotics
- Extent of IV to oral switching (based on the stability of the gastrointestinal tract)
- Duration of surgical prophylaxis
- Changes in antibiotic use over time in terms of Defined Daily Doses/100(0) bed days and/or days of therapy/patient-days
- Whether active antimicrobial stewardship groups and/or drug and therapeutic committees/Infection Prevention and Control groups in the hospital

Multiple activities to reduce HAIs include encouraging greater hand hygiene, regular reviews surrounding the use and care of catheters, instigating appropriate strategies to reduce ventilator-associated pneumonia including subgluteal space suctioning before each position change, chlorhexidine gluconate bathing as well as active monitoring of agreed guidelines to reduce HAIs [132,221,222]. A number of initiatives have been undertaken to improve hand hygiene in LMICs including those orchestrated by the WHO and others providing direction [223,224].

Multiple activities can also be undertaken to improve antibiotic utilization to reduce SSIs among LMICs [84]. Recent publications have documented that the number of patients receiving their first dose of antibiotics within 60 min of the first incision, with a second dose administered for longer procedures, can be as low as 6.7% of surgical patients in LMICs [84,108,225,226]. This is a concern as studies have shown the risks of SSIs are almost five times higher when antibiotics are administered more than two hours prior to the first incision and almost doubled when administered after the first incision [84,227]. There are also concerns that extending prophylaxis beyond one day, which is frequent among LMICs with for instance a mean up to 8.7 days post-operatively in Nigeria, does not appear to improve outcomes whilst concurrently increasing the potential for acute kidney injury, *Clostridium difficile* infections, and AMR as well as increasing costs [84,228,229,230,231,232,233]. Vicentini et al. (2019) in their published study found that adequate antibiotic choices and length of administration were associated with significantly reduced risks of SSIs [234], with a number of approaches successfully undertaken in LMICs to improve antibiotic use surrounding the prevention of SSIs [84].

#### 3.1.1. Antimicrobial Stewardship Programs (ASPs) and Other Activities to Improve Antimicrobial Utilization in Hospitals

ASPs as well as other interventions can improve overall utilization of antimicrobials in hospitals as well as reduce costs alongwith improved hand hygiene and other activities to reduce HAIs and SSIs (Table 1) [223,235,236,237]. There are a number of areas that can be initial targets for ASP programs to gain their acceptance among key stakeholder groups in hospitals to address barriers [211,212,213]. These include developing guidelines for specific indications and monitoring subsequent usage against these guidelines, as well as developing prompts for physicians to regularly review antibiotic prescriptions.

Table 2 summarizes some of the activities that have been undertaken in LMICs to improve antimicrobial utilization in hospitals, which build on reviews in predominantly high-income countries including the use of computerized decision support systems [236,238]. This includes (Table 2) activities surrounding SSIs and their impact to provide future guidance. Activities principally centre on educational initiatives [93], with educational initiatives known to enhance understanding of ASPs in LMICs given concerns about knowledge and activities among students and physicians in a number of LMICs [214,215,239,240].

We are aware that ASPs have been successfully instigated in Accident and Emergency Departments in hospitals. However, this typically applies to higher-income countries [134,260,261].

#### 3.1.2. Dealing with Antimicrobial Shortages in Hospitals

Other strategies that antimicrobial stewardship groups and DTCs can undertake in hospitals is to develop effective approaches to address antimicrobial shortages, with medicine shortages being an increasing concern across countries [145,262,263,264]. Shortages of antimicrobials can lead to administering less effective, more toxic, and costlier antimicrobials as well as potentially increasing AMR rates [264,265,266]. One way to address this is for key personnel in hospitals, including those who are part of ASTs, to agree in advance therapeutic interchange strategies [264]. This can include consultations between prescribers and hospital pharmacists before alternative antibiotics are administered [264].

Emergency procurement procedures should also be in place in hospitals alongside regular reviews of stock levels in wards, exacerbated by the current COVID-19 pandemic [263,267]. Available resources and funding is also essential to reduce the possibility of antimicrobial shortages in hospitals. The Bamako Initiative in sub-Saharan Africa is one such initiative adopted by Ministers of Health to increase access to medicines including antimicrobials through revolving drug funds [268,269]. However, sustaining earlier gains with this initiative remains a challenge, and alternative drug financing schemes are essential to ensure physicians and patients can access appropriate antimicrobials in hospital to improve outcomes and reduce length of stay [270].

### 3.2. Antimicrobial Use in Ambulatory Care

We will principally concentrate on ARIs as these are the most common infections seen in ambulatory care in LMICs and where the highest rate of inappropriate prescribing and dispensing takes place [64,102,271,272,273]. Suboptimal management of ARIs in LMICs is exacerbated by a number of issues including social-cultural issues, diagnostic uncertainty, clinical competency and misconceptions as well as from commercial, patient and time pressures [64,114,129,133,274,275,276].

Consequently, there are a number of strategies that can be undertaken in ambulatory care across LMICs to address current issues to improve future antimicrobial utilization. These can principally be divided into those impacting on the prescribing of antimicrobials by physicians for what are essentially viral infections, those strategies that can reduce inappropriate dispensing of antibiotics without a prescription as well as those that can address concerns with multi-resistant organisms for infections such as malaria and TB. In addition, there are a number of strategies that can be implemented to improve vaccination rates including during pandemics.

Improving antimicrobial utilization in ambulatory care for ARIs is especially important in the current pandemic, with, as mentioned, published studies suggesting that over 70% of patients with COVID-19 are receiving antibiotics, including broad-spectrum antibiotics, even when not clinically indicated [51,52], with perhaps as low as 3.2% of infections in ambulatory care warranting antibiotics [21,54].

#### 3.2.1. Initiatives among Physicians to Improve Antibiotic Prescribing

A number of initiatives have been undertaken across LMICs to improve physician prescribing of antibiotics for ARIs. Table 3 summarizes examples of the many multiple activities that have been undertaken in LMICs to improve antimicrobial prescribing especially for ARIs, principally in education, and their impact.

Among LMICs, there is an absolute imperative to develop actionable quality and patient safety indicators that better represent current prescribing compared with the traditional WHO/INRUD criteria [284]. This reflects the rise in the number of patients in LMICs, especially sub-Saharan Africa, with both infectious and non-infectious communicable diseases (NCDs) as well as combinations of infectious diseases such as HIV and TB and combinations of infectious diseases and NCDs [285,286,287,288,289]. Consequently, there is a need to develop more specific quality and patient safety indicators for LMICs to improve future antimicrobial prescribing. This can be achieved using agreed and common coding of data to enable coordinated and transferable learning between countries including patients attending ambulatory care clinics in sub-Saharan Africa. This will be the subject of future research activities among the group.

These activities build on recent studies, including those undertaken in sub-Saharan Africa, suggesting that adherence to guidelines is a better indicator of the quality of prescribing in ambulatory care in LMICs compared with the current WHO/INRUD criteria [167,169,181,290]. Having said that, it is important that there is only one national reference guideline since contradictions can arise when there are multiple guidelines within a country [291]. It is also essential that any guidelines produced must be readily available in multiple media formats including online and downloadable, as well as in print. National guidelines also need to be combined with educational inputs and auditing of prescribing practices to further improve future prescribing [114,172,181,290,292,293].

Regular updates of any guidelines, especially online, are also essential to improve adherence rates, and any updates need to be rapidly disseminated. This is being achieved among a number of sub-Saharan African countries through mobile technologies [101,293], providing guidance to other LMICs.

We are aware though that over prescribing of antibiotics can be appreciably more common in telemedicine consultations than in face-to-face visits [294,295]. Consequently, these consultations also need to be part of any future quality initiatives.

#### 3.2.2. Initiatives to Reduce Inappropriate Dispensing of Antimicrobials among Pharmacists in the Community

There are concerns with the extent of inappropriate dispensing of antimicrobials in community pharmacies and drugs stories across LMICs, especially with, as mentioned, such utilization accounting for up to 93% to 100% of all dispensed antibiotics [123,124,129,135,136].

These concerns can be exacerbated by variable knowledge regarding antibiotics and AMR among community pharmacists [162,296,297,298]. Having said this, there are key issues in rural settings in LMICs that need to be considered by Governments and others when reviewing strategies to reduce inappropriate dispensing of antimicrobials. These include the fact that pharmacists maybe the only healthcare professional available and they are trusted [49,135,156,299]. There can also be major issues with affordability among patients in LMICs. Meeting both physician fees and the cost of medicines can have catastrophic consequences for individuals and families in LMICs [151,152,300]. Trained pharmacists, assisted by guidelines, can reduce inappropriate self-purchasing of antibiotics in LMICs as well as be leaders of ambulatory care ASPs in their countries to improve future antibiotic utilization especially for infections that are essentially viral [99,157,301].

Strategies to successfully reduce inappropriate self-purchasing of antimicrobials range from having well-educated pharmacists offering advice (Education) to patients as seen in Kenya, regulations by law restricting the purchasing of antimicrobials without a prescription coupled with fines (Economics and Enforcement), or a combination of these as seen in the Republic of Srpska (Education, Economics and Enforcement) [99,100,157,183].

The extent of ‘enforcement’ including the potential for fines or closure of pharmacies is vital as this impacts on the outcome of this intervention (Table 4). For instance, in Venezuela, the Government implemented policies to limit self-purchasing of antibiotics of three antibiotic groups. However, there were no public awareness campaigns and ‘Enforcement’ was only via government publications with no follow up. As a result, there was no decrease in antibiotic utilization levels but, in fact, the opposite with an increase [302]. Similarly, in Colombia, whilst initial enforcement of the law in 2005 had a modest impact on overall sales in the first three years (−1.00 DDDs/1000 inhabitants per day), a follow-up study five years after implementation found that 80.3% of pharmacies were still not complying with the law due to lax monitoring, prompting calls for greater enforcement [302,303]. This contrasts with an appreciable reduction in antibiotics dispensed without a prescription in a number of countries following multiple interventions, e.g., Saudi Arabia (Table 4).

We see similar examples in other disease areas where the intensity of follow-up of prescribing restrictions (enforcement) appreciably impacts on their effectiveness and outcome in practice [304,305,306].

However, it can be difficult in practice in a number of LMICs to enforce regulations banning self-purchasing of antibiotics due to manpower issues as well as shortages of antimicrobials within public healthcare facilities. Such activities may also be counter-productive in rural areas in LMICs and we have seen that trained pharmacists can help reduce inappropriate dispensing of antibiotics.

Monitoring pharmacy dispensing activities through mobile technologies as well as potentially implementing information technology (IT) surveillance systems to track antibiotics through the supply chain are also potential strategies to reduce inappropriate dispensing [99,135].

Adoption of such tactics may mean re-looking at pharmacists’ knowledge regarding AMR and its causes, with the potential for increased training if needed starting in pharmacy school and continuing post-qualification [159,162,314,315]. In addition, potentially ensuring through regulation and other mechanisms that any antibiotic dispensed for ARIs and other common conditions are principally from the Access group of antibiotics and not from the WHO ‘Watch’ or ‘Reserve’ list [173,174].

#### 3.2.3. Initiatives to Reduce Inappropriate Dispensing of Antimicrobials among Patients and the Public

The impact of programs to reduce inappropriate prescribing and dispensing of antimicrobials for essentially viral infections such as ARIs can be enhanced by concurrently targeting patients and the public. This is because multiple studies have shown that some people can put considerable pressure on healthcare professionals to prescribe and dispense antibiotics for ARIs [314,316,317,318]. Knowledge about antibiotics and AMR can vary though among patients and the public, and they can have strong beliefs about the effectiveness of antibiotics for them even for viral infections, which adds to the pressure to prescribe or dispense antimicrobials for ARIs [318,319,320,321,322,323,324].

Similar to the situation with physicians and pharmacists, multifaceted approaches are needed to change attitudes and behaviors among patients and caregivers [325,326]. However, such programs have had variable degrees of success for countries including both high-income countries and LMICs [318,327,328,329]. Published studies, particularly among high-income countries, suggest that a number factors can enhance the success of any antibiotic awareness campaign. These include (i) the use of carefully designed simple key messages; (ii) targeting a wide audience including patients and their families; (iii) designing key messages for all key stakeholder groups for completeness; (iv) using mass media including social media to raise awareness; and (v) repeating key messages especially for new parents and others [318,328,330,331,332].

Two systematic reviews have suggested that campaigns among patients can significantly reduce antibiotic utilization. McDonagh et al. (2018) found that parent education reduced antibiotic prescribing for ARIs by 21% [116]. Cross et al. (2017), mainly including studies from high-income countries, also found that the majority of studies reported reductions in antibiotic utilization. The reduction was greater than 14% in the prescribing of antibiotics for RTIs, up to a maximum reduction of 30% [318].

However, the impact of patient education initiatives in LMICs have not yet been fully elucidated, nor their costs and cost-effectiveness [115]. Consequently, caution is still needed before fully embarking on such campaigns in LMICs unless these are part of research programs.

#### 3.2.4. Initiatives to Address MDR Organisms for HIV, Malaria and TB

There are concerns that patients with TB living in rural areas, and without regular supply of medicines, will have poorer treatment outcomes [80,333]. Potential reasons for this include insufficient funds for transport to treatment centers despite the medicines often being provided free of charge alongwith missing out on regular reviews of medicine use addressing concerns including compliance with prescribed medicines, similar to the situation with NCDs [80,158,334]. Poorer outcomes and lack of adherence to treatments will enhance resistance rates [80,335].

The overall burden of TB has been declining at an annual average of 2%; however, the number of patients with drug-resistant TB (DR-TB) is increasing. Aminoglycosides, such as kanamycin, and some polypeptides, such as capreomycin, are used to treat DR-TB. However, whilst aminoglycosides are inhibitors of prokaryotic protein synthesis at commonly accepted therapeutic concentrations, they can affect the protein synthesis of cells at larger concentrations, leading to toxicity such as ototoxicity, vestibulotoxicity, and nephrotoxicity [336]. Hearing loss though can now be monitored using an ototoxicity grading system within a mobile app to assist health professionals in assessing patients for ototoxicity. Subsequently, monitoring progression of hearing loss to improve planning for any changes in care [337]. Such developments are likely to continue to improve the future use of aminoglycosides.

There are also concerns with rising resistance rates to antimicrobials used to treat malaria since for instance when resistance of *P. falciparum* to sulfadoxine-pyrimethamine was greater than 10%, the risk ratio for malaria infections was 5.9 times higher compared to a period of time with low resistance rates, and the risk of death from malaria 10.8 times higher [6,338].

Developing resistance to treatments for HIV also increases mortality with studies from sub-Saharan Africa indicating that when pre-treatment drug-resistance levels are evident but below 10%, AMR will be responsible for an estimated 710,000 AIDS deaths and 380,000 new infections by 2030. This increases to an estimated 890,000 AIDS deaths and 450,000 new infections by 2030 attributable to HIV when resistance rates are above 10% [6,339].

Strategies to address concerns with resistance to treatments for HIV, malaria and TB include initiatives to ensure regular access to treatment facilities, including expanding these in rural areas given concerns with travel costs and time off work, as well as regular access to treatments ideally provided free of charge. Alongside this, regular monitoring of adherence rates knowing adherence can be a major issue in some patients [80,340,341,342]. Strategies to improve adherence rates include patient and staff education addressing pertinent issues of anxiety and depression with their condition, encouraging clinic visits, addressing socioeconomic factors where possible as well as increasing the use of digital technologies including mobile phones and video-observed therapy [341,342,343,344,345,346,347,348].

Concurrent with this, we are aware of groups in South Africa undertaking research on HIV reservoirs and using locally discovered potent, broadly neutralizing antibodies to neutralize HIV in the reservoir to render patients non-infectious as a cure strategy [349]. Recent promising data from primates indicate the potential value of this approach and clinical trials are being planned.

We are also aware of ongoing research that provides an expansive dataset of compounds that could be redirected for antimalarial development and also point towards proteins that can be targeted in multiple parasite life cycle stages to improve the management of patients with malaria resistant to current treatments [350]. We will be monitoring these developments and their implications.

#### 3.2.5. Programs to Address Concerns with Vaccine Uptake Including Misinformation as Well as Other Situations Adversely Affecting Antimicrobial Use

Reduced vaccine uptake including vaccine hesitancy fueled by misinformation is a concern in view of the impact on AMR, morbidity and mortality (Table 5) [10,37].

This increasing recognition of the role of vaccines in reducing antimicrobial use and AMR is reflected in the recent WHO Action Framework to enhance current vaccination rates, encourage greater knowledge and dissemination on the role of vaccines to reduce AMR as well as encourage the development of new vaccines to further reduce AMR [358,359].

Addressing mis- and dis-information surrounding vaccines is critical to reduce future morbidity and mortality arising from pandemics [360,361]. We are already seeing social media companies such as Facebook and Twitter tightening up on accounts that repeatedly publish misinformation about COVID-19, building on activities among some African countries to fine companies for spreading misinformation [36,362], and such activities are likely to continue. This builds on activities by the WHO to join forces with Governments to address the level of misinformation surrounding COVID-19 as an example [363] as well as Governments funding projects to specifically address misinformation surrounding vaccines for COVID-19 to enhance their uptake [364,365]. Inclusivity is essential to engage with community groups and patient organizations [366], and ensure that clear public health messaging is delivered that is sensitive to age, culture, socioeconomic context and education, and to build population resilience against AMR behaviors [367].

Potential ways forward, building on the activities of the WHO and Governments, especially with proposed medicines for COVID-19 including hydroxychloroquine, lopinavir/ritonavir and remdesivir that failed to live up to their preceived promise [368,369,370,371,372,373], include instigating a culture of evidence-based medicine among all key stakeholder groups starting in universities and continuing post-qualification [36,166,374]. Such activities would have helped reduce shortages and price rises of hydroxychloroquine and antibiotics arising from the initial studies as well as help reduce inappropriate prescribing of antibiotics [41,49,153,166].

Governments and others working with pertinent patient organizations within countries also need to proactively address misinformation [366], especially if, as mentioned, this increases future morbidity, mortality and costs, as seen with misinformation regarding hydroxychloroquine and unwarranted fears about vaccines. Transparent communication and appropriate public health messaging, in collaboration with patient groups and community leaders, are key for increasing the trust of the community and improving vaccination rates where concerns.

### 3.3. Suggested Activities among All Key Stakeholder Groups to Improve Future Antibiotic Utilization

Suggested strategies to improve the utilization of antimicrobials among LMICs and thereby reduce AMR have been divided into short- and long-term initiatives to provide future guidance (Table 6). These build on the potential for expanding the use of electronic health records and electronic prescribing as well as digital technologies, including clinical decision support systems (CDSS), to improve appropriate use of antibiotics across countries and sectors in LMICs [101,238,375,376]. Such developments should be welcomed given the increasing complexity of medical decision making and, as mentioned, the high percentage of patients especially in sub-Saharan Africa with co-morbidities including HIV, malaria and TB, as well as co-morbidities with both infectious and non-infectious diseases.

Effectively addressing inappropriate antibiotic prescribing is particularly challenging since the majority of antibiotic prescribing in LMICs is undertaken by general clinicians and their assistants who are not experts in infection management and with often influence from pharmaceutical companies in the absence of continual professional development [155,172,377,378,379]. This is compounded across Africa due to high workloads as well as limited consultation times and available resources, driven mainly by a shortage of healthcare professionals and the high prevalence of infectious diseases [380]. Recent evidence is promising though about the feasibility and effectiveness of digital tools to improve future antibiotic prescribing and dispensing [101,204,205,381].

## 4. Discussion

AMR continues to be a high priority across countries in view of its impact on morbidity, mortality and costs [11,15,19], driven by the overuse of antimicrobials across all sectors including in animals and for the treatment of infectious diseases in patients. The overuse of antimicrobials in humans has been exacerbated by the recent COVID-19 pandemic, where, as mentioned, typically less than 10% of COVID-19 patients have bacterial or fungal infections [53], with as low as 3.2% of COVID-19 infections warranting antibiotics [21,54]. There are similar concerns with current low vaccination rates in a number of countries including current vaccine hesitancy surrounding the COVID-19 vaccine alongwith misinformation surrounding treatments for COVID-19, all of which negatively impact on costs and outcomes [36,43,49,153,161,166,360].

Despite issues of resources including personnel, a number of activities can be undertaken in LMICs to improve future prescribing of antibiotics. These center around the instigation of ASPs in hospitals including PPS studies to identify areas for quality improvement, with a number of quality indicators now developed to monitor the appropriateness of future prescribing (Box 1). Strategies to improve future antibiotic prescribing in hospitals include those to reduce inappropriate timing and duration of antibiotic prescribing to prevent SSIs. Table 1 and Table 2 document a number of examples of initiatives that have been successfully instigated in hospitals in LMICs to improve future antibiotic prescribing, thereby providing exemplars to others.

Successful strategies have also been implemented among physicians in LMIC to reduce inappropriate prescribing of antibiotics for essentially viral infections (Table 3), with patients playing a key role. Patients and pharmacists are also important to reduce inappropriate dispensing of antibiotics without a prescription (Table 4). However, recognizing that in rural LMICs, especially where there are high patient co-payment levels, community pharmacists maybe the only healthcare professional accessible and available. Community pharmacists are often more accessible than ambulatory care physicians in LMICs, and there is no need for physician co-pays, which combined with the cost of treatments can be catastrophic for some families [151,152,300].

Healthcare professionals, including community pharmacists, along with patient organizations have a key role to play to encourage evidence-based approaches to healthcare, thereby reducing the impact of misinformation. Misinformation has been a real concern during the current COVID-19 pandemic resulting in for instance reduced uptake of the vaccines and the implications for this and other vaccines (Table 5), with a number of strategies identified to address this (Section 3.2.5).

A number of strategies and initiatives have also been identified to reduce levels of multi-drug resistance organisms to HIV, malaria, and TB, given the resultant impact on morbidity and mortality. Key areas include the provision of treatments free of charge, ready access to healthcare facilities as well as initiatives to enhance adherence to prescribed treatments.

Potential strategies for all key stakeholders were subsequently consolidated into short-, medium- and long-term activities to provide future direction (Table 6). The key is Government commitment through NAPs and other activities to drive forward future initiatives. We will be monitoring the situation given continued concerns with AMR and its resultant impact on mortality as well as GDP.

We are aware of a number of limitations with this review paper. These include the fact that we did not undertake a systematic review for the reasons discussed. We also did not break the number of examples down by country or care setting as our objective was to provide pertinent examples to guide others. In addition, we are aware that some developing countries are more pro-active with their research than others, potentially biasing the findings. Despite these limitations, we believe that our findings and suggestions are robust, providing future direction.

## 5. Conclusions

In conclusion, reducing AMR should be a high priority across countries given its clinical and economic impact. Reducing AMR rates requires multiple coordinated activities across sectors driven by Governments and others. This is essential given limited new antimicrobials being developed, although compensated to some extent by developments in vaccine technologies. This will also require strategies to address high rates of vaccine hesitance that exist in a number of countries as seen in the recent COVID-19 pandemic. A coordinated approach including all key stakeholder groups is also essential to minimize misinformation and maximize the impact of future interventions to reduce AMR rates.

## Figures and Tables

**Table 1:** Hospital activities to improve antimicrobial use.

Activity	Role with Improving Antimicrobial Prescribing
IPC committees	Can be a core component of enhancing antimicrobial use within hospitals through instigating pertinent quality improvement programs [189], with minimum requirements for IPCs established by the WHO and others [190,191] Studies have shown that IPCs can reduce AMR in key hospital wards [191] However, there can be issues with lack of resources and identity as well as negative staff attitudes [187]
PPS studies	PPs studies are important to gain an understanding of current antimicrobial use in hospitals and potential programs for quality improvement initiatives [105,168,193,194] PPS studies help determine the extent of HAIs within LMICs [104], with typically a higher rate of HAIs among LMICs at 5.7–19.1% of patients versus 5.7–7.5% in high-income countries [104,195,196]—although rates up to 28% to 45.8% have been recorded among hospitals in sub-Saharan African countries [104,197,198] This is a key issues as HAIs are associated with appreciable morbidity, mortality and costs [104,131,199,200], with higher prevalence rates of HAIs in LMICs due to a number of factors including poorly enforced ward environmental sanitation practices, lack of monitoring of adherence to IPC guidelines and concerns with facilities [104,201] Key concerns with PPS studies include the extent of documentation of the rationale for the choice of antimicrobial(s), whether the rationale is in line with local/national recommendations, whether culture and sensitivity testing (CST) is being routinely undertaken, the extent of switching from intravenous (IV) to oral use, the timing and duration of administration of antibiotics to prevent SSIs and adherence to local or national guidance—increasingly seen as a key quality indicator [88,168,169,171,202] Specific data collection forms have been designed for Africa recognizing the additional burden of HIV, TB, malaria and malnutrition [88,203], with APPs developed to accelerate data collection and analysis [204,205]
ASPs	Seen as a core element especially within hospitals to improve future antibiotic prescribing (Section 3.1.1) Publications including from the Commonwealth Partnership for Antimicrobial Stewardship and practical toolkits from the WHO and others can assist with implementation [171,206,207,208,209,210,211] However, ASPs can take time to implement in LMICs in view of ongoing cultures and resources [97]: ○ In Tanzania, one year after the instigation of the NAP, 35.9% of respondents had instigated some form of ASP in their hospital; however, hospital antibiotic policy documents were only available in 15.4% of facilities [76]. Encouragingly, IPC committees were now present in the majority of hospitals surveyed ○ In Vietnam, by the end of 2018, ASPs had been initiated in 47% of its hospitals, but actual implementation has been slow [212,213]

**Table 2:** Summary of published studies across LMICs documenting the impact of ASP interventions including those surrounding SSIs.

Author, Country and Year	Brief Details of the Intervention	Impact of the Intervention
Bozkurt et al., Turkey, 2014 [241]	Principally educational interventions to improve appropriate antibiotic use for SSIs. These included: Series of meetings with physicians from each clinic organized by the Infection Control Committee Daily visits from the Infection Control Nurse and twice weekly visits from the Infectious Diseases Control Specialist—more if compliance with agreed guidelines was low Observations regularly shared with the group	Use of appropriate antibiotics increased from 51% to 63.4% of cases Duration of antibiotic use improved from 10.3% to 59.4% of cases Total cost of antibiotics in the medical units, surgical units and ICUs decreased by 32.5%, 38.6% and 11.1%, respectively, over the time of the study
Hou et al., China, 2014 [242]	A number of interventions were undertaken to improve antibiotic utilization in ICUs as part of ASPs. These included: Education—all healthcare professionals trained on antimicrobial-stewardship relevant knowledge as well as developing a new antibiotic formulary Engineering—Physicians had strict regulations surrounding antibiotic prescribing including restrictions surrounding quinolones. Target of 40 DDDs/100 patient-days for the hospital and 120 for the ICU, with regular feedback to the Director of the Unit	Total antibiotic consumption in the hospital decreased from 69.69 DDDs/100 patient-days to 50.76 DDDs between April and August 2011; and in the ICU decreased from 197.65 to 143.41 DDDs/100 patient-days Significant improvements in resistance to amikacin, ceftazidime, ciprofloxacin, ceftriaxone, gentamicin, ofloxacin and piperacillin in *Enterobacteriaceae* and resistance to ceftazidime, imipenem, and meropenem in non-fermenting Gram-negative rods Rates of no prescribing of antibiotics initially or just a single antibiotic prescription significantly increased (*p* < 0.001)
Amdany et al., Kenya, 2014 [243]	Principally an educational initiative to enhance the use of oral vs. IV metronidazole including education, audit and feedback	Post-implementation audit showed an increase of more than 40% compliance in all the four criteria utilized to assess an increase in oral use As a result, reduced costs, patient discomfort and possible iatrogenic infections
Yang et al., China, 2014 [244]	Education and Engineering interventions to improve antibiotic use for SSIs. These included the Introduction of a Drug Rational Usage Guideline System (DRUGS) vs. paper-based guidelines to enhance adherence to surgical prophylaxis guidelines	Timing of the initial dose improved from 32.9% of patients with antibiotics instigated within 30 min to 2 h pre incision to 85.8% post-intervention (statistically significant) Average length of stay decreased from 7.00 days with paper-based guidelines to 2.55 days with DRUGS Average cost of antibiotics prescribed decreased from ¥3481 with paper-based guidelines to ¥1693 with DRUGS
Okumura et al., Brazil, 2015/2016 [245,246]	Primarily Education comparing the outcomes from two different ASP approaches among patients in the general ward and ICU: Bundled ASP including clinical pharmacist chart review, discussions with microbiologists/infectious disease physicians, education of physicians and continuous follow-up of pertinent physicians until clinical resolution/discharge Conventional ASP involving a clinical pharmacist chart review and discussions with infectious disease physician	DDDs/1000 patient-days decreased from 557.2 to 417 (*p* < 0.05) in the bundled ASP group Mortality decreased in the bundled ASP group (*p* < 0.01) and the risk difference was 10.8% (95% CI: 2.41–19.14) The bundled strategy though was overall more expensive at US$2119.70/patient; however, more cost-effective
Saied et al., Egypt, 2015 [247]	Principally Education and Engineering to improve antibiotic use of SSIs. These included 2 day training curriculum course for pertinent surgeons On-the-job training provided to junior surgeons and residents Wall-mounted poster developed to remind prescribers of the optimal timing and duration of antibiotic administration for SAP Regular audit and feedback meetings orchestrated by the senior surgeon in the hospital (3 of 5 participating hospitals)	The optimal timing of the first dose improved significantly in the 3 hospitals subject to the intervention—increasing from 6.7% of pertinent patients to 38.7% (*p* < 0.01) All hospitals involved showed a significant rise in the optimal duration of surgical prophylaxis—overall increase of 3–28% across the hospitals (*p* < 0.01)
Ntumba et al., Kenya, 2015 [248]	Principally Education and Engineering to improve the use of antibiotics in relation to SSIs. These included: Local adaptation of guidelines Creation and tools for advocacy, training, and leadership around appropriate SAP	Patients receiving antibiotics post-operatively decreased from 50% to 26% Crude SSI rates significantly decreased from 9.3% to 5% of patients
Apisarnthanarak et al., Thailand, 2015 [249]	This ASP principally involved Education with a 12 h training course run by infectious diseases clinical pharmacists (IDCPs) with physicians looking after patients in medical wards coupled with an option for infectious diseases consultations (IDCs), daily rounds with ICDPs, or both if wished, with a control (Usual Standard of Care) group	For patients with input from the IDCP group or the IDCP plus IDC group vs. controls, they were: Less likely to be prescribed inappropriate antibiotics (*p* < 0.001) Have greater de-escalation of antibiotics (*p* < 0.001) Received antibiotics <7 days (*p* < 0.001) Have shorter lengths of stay (*p* < 0.001)
Boyles et al., South Africa, 2017 [250]	Principally Education over a sustained period for this ASP Intervention comprising online education materials, a dedicated antibiotic prescription chart and weekly dedicated ward rounds	Total antibiotic consumption fell from 1046 DDDs/1000 patient-days in 2011 (control period) to 868 by 2013 and remained at this level for the next 2 years—driven by a reduction in IV antibiotic use, particularly ceftriaxone Inflation-adjusted cost savings on antibiotics were SA Rand of 3.2 million over 4 years
Brink et al., South Africa, 2017) [251]	Education and Engineering. Key activities driven by hospital pharmacists included: Recording current SSI rates and developing a SAP ‘toolkit’ Testing and revising the SAP guidelines and toolkits at pilot sites prior to their launch at regional training and institutional workshops Obtaining consensus and endorsement from key professionals in the hospital through adapting and modifying guidelines where appropriate Choosing at least one or more surgical procedures to audit including recording pre-intervention SAP practices and trends for the chosen surgeries Subsequently measure compliance to agreed four process measures over a 4-week period and feedback	Timely administration of antibiotics increased to 56.4% of surgical patients (*p* < 0.0001) Antibiotic choice consistent with the guidelines increased to 95.9% of patients and the duration of prophylaxis was now appropriate among 93.9% of patients
Allegranzi et al., Kenya, Uganda, Zambia, and Zimbabwe, 2018 [252]	Principally Education and Engineering to improve antibiotic prescribing for SSIs. This included: 5 planned visits to each hospital in the four African countries during the study period supported by a range of educational tools Local teams identified key areas of concern with preventing SSIs to concentrate on through monitoring an agreed range of SAP indicators (six pre-identified ones including skin preparation and optimal timing of prophylaxis) Subsequent launch activities of pertinent tools and indicators alongside monitoring/feedback to improve prescribing	Appropriate use of SAP improved from 12.8% (baseline) to 39.1% of patients (*p* < 0.0001) among the studied hospitals Cumulative SSI incidence decreased from a baseline of 8.0% to 3.8% post-intervention (*p* < 0.0001)
Khdour et al., Palestine, 2018 [253]	Principally education—the ASP team drew up new antibiotic guidelines for the empiric treatment of common infections within the ICU Clinical pharmacists subsequently performed the initial review of subsequent treatment and made therapeutic recommendations if needed which were reviewed by the ASP team on the 2nd, 4th and 7th days, allowing for CST findings. Subsequent interventions were recommended by the ASP team if needed Four months of pre-ASP data were compared with 4 months of post-ASP data to assess the impact	High acceptance of ASP team recommendations (78.4%)—most accepted were dose optimization (87.0%) and de-escalation (84.4%) ASP interventions significantly reduces antimicrobial use—from 87.3 DDDs/100 beds vs. 66.1 DDDs/100 beds *p* < 0.001) Median length of stay was significantly reduced post-ASP—down from 11 (3–21) to 7 (4–19) days (*p* < 0.01)
Abubakar et al., Nigeria, 2019 [254]	Principally Education and Engineering to improve antibiotic prescribing for the prevention of SAP. This included: Development and dissemination of an agreed departmental protocol for SAP, presented and agreed before its adoption to enhance subsequent adoption rates Educational meetings with key clinicians to enhance uptake of agreed protocols combined with wall mounted posters Regular audit and feedback meetings using baseline data	Patients in the postintervention period were 5.6 times more likely to receive SAP within 60 min before the incision vs. pre-intervention (*p* < 0.001) The rate of redundant antibiotic prescriptions was reduced by 19.1%
Karaali et al., Turkey, 2019 [255]	Multiple activities to improve antibiotic prescribing for SSIs including Education, and Engineering. These incorporated: Local guidelines updated by two members of the infection control committee One general surgery team leader appointed to be responsible for improving SAP as part of ASPs in their group Periodic training sessions to supervise and regulate SAP by surgical team leaders Instigation of a new protocol, whereby clean and clean-contaminated cases would not be given SAP for longer than 24 h and that discharge prescriptions would not include antibiotics. However, no verbal or written sanctions were imposed for abuse	Compliance with SAP guidance significantly increasing from 55.6% to 64.5% of patients (*p* < 0.05)—although differences between surgical types Significant reduction in the extent of prolonged prophylaxis (beyond 24 h) from 60.2% of patients pre intervention to 7.5% post-intervention (*p* < 0.05) Extent of antibiotic prescribing after discharge reduced to 9.4% of patients down from 80.6% of patients pre-intervention (*p* < 0.05) However, limited impact on the timing of first antibiotic dose—increasing from 81.9% of patients to 83.7%
Mahmoudi et al., Iran, 2019 [256]	Principally Education and Engineering to improve SAP: Revising SAP guidelines following meetings between a clinical pharmacist and the surgical department with senior clinical pharmacists delivering lectures about SAP to key members of the surgical departments Clinical pharmacists participating in ward rounds, attending recovery rooms and communicating with surgeons when guidelines not followed Clinical pharmacists providing educational materials Rationality of SAP continually evaluated during the perioperative period in accordance with agreed guidelines with clinical pharmacists communicated any concerns directly with relevant physicians	Rate of antibiotic prescribing beyond 48 h appreciably improved to just 5.7% of patients—down from 92.1% of patients pre-intervention Appropriateness of antibiotic use increased to 91.4% of patients from 30.1% pre-intervention Mean cost of antibiotics decreased more than 11-fold and length of stay decreased from an average of 5.14 days pre-intervention to 4.33 days (*p* < 0.001) post-intervention
Xiao et al., China, 2020 [257]	Multiple activities include Education, Engineering, Economics and Enforcement over a 6-year campaign: Hospitals were encouraged to establish ASTs with healthcare authorities promoting education and training programs for medical staff Health authorities also supervised hospitals and issued penalties including personal warnings and dismissals, as well as downgraded hospitals Prescription rights were withdrawn where serious protocol violations observed	Over the six years—2016 vs. 2010: Proportion of outpatients and surgical patients who received antibiotics decreased from 19.5% to 8.5% and from 97.9% to 38.3% respectively Overall antibiotic use decreased from 85.3 ± 29.8 DDDs/100 patient days to 48.5 ± 8.0 Antibiotic procurement expenditure declined from 22.3% of total drug procurement costs to 12.1% Incidence of methicillin-resistant *Staphylococcus aureus* isolates decreased from 54.4% to 34.4% in 2016); similarly, for carbapenem-resistant *Pseudomonas aeruginosa* isolates from 30.8% to 22.3%
Mardani et al., Iran, 2020 [258]	Principally education for this ASP comprising: Continuous educational programs for nurses and physicians employed in different wards of the hospital An inter-disciplinary ASP team performed a weekly scrutiny of treatment for patients based on their electronic medical records and provided feedback include reduction in multiple antibiotic use and dosage changes	Reduction in MDR cases in the year post-intervention from 145 and 75 (*p* = 0.011) Significant reduction in all positive blood cultures (*p* = 0.001) Significant reduction in meropenem use (*p* = 0.043) as well as a significant reduction generally in antibiotic consumption, multidrug-resistant organisms and CDIs
van den Bergh et al., South Africa, 2020 [259]	Principally education to improve compliance to agreed guidelines: A Community Acquired Pneumonia (CAP) bundle was developed including seven process measures (including admission criteria, drug choice, dose and length) and three outcome measures (including length of stay and mortality) that pharmacists used to audit compliance to the bundle and provide feedback Training sessions were conducted on the CAP and implementing ASPs within hospitals. After each learning session, a checklist of essential activities and deadlines was provided to each pharmacist Baseline data were collected to identify areas for improvement. In a four-week period after the learning sessions, pharmacists applied the learnt ideas to improve compliance to the CAP guidelines and ways to give feedback to address identified gaps to improve future compliance with multi-disciplinary team members and hospital leadership	2464 patients from 39 hospitals were included with the ASP showing positive results: CAP bundle compliance improved to 53.6% from 47.8% to 53.6% (*p* < 0.0001) Diagnostic stewardship compliance improved to 54.6% from 49.1% of patients (*p* < 0.0001) Improved compliance with process measures was significant for five of the seven components. These include choice and dose of antibiotics prescribed as well as IV to oral switch However, there was no significant difference in mortality or median length of stay pre- and post-intervention The study represents the first collaboration between public and private sector hospitals in ASPs in South Africa

**Table 3:** Initiatives to improve antibiotic utilization among physicians in ambulatory care in LMICs especially for ARIs.

Author, Country, Year	Intervention and Impact	Impact
Teng et al., Malaysia, 2006 [277]	Education—Academic detailing from the resident family medicine specialist accompanied by an information leaflet	Reduction in general antibiotic prescribing rates from 14.3% pre-intervention to 11.0% post-intervention (RR 0.77, 95% CI 0.72 to 0.83) Reduction in URTI-specific antibiotic prescribing rates from 27.7% and 16.6% post-intervention (RR 0.60, 95% CI 0.54 to 0.66).
Shrestha et al., Nepal, 2006 [278]	Principally Education—7 health posts and 33 subhealth posts were stratified by type with health workers. The intervention was based on 5 days of training on the adapted Practical Approach to Lung Health (PAL) guidelines and their use	Appropriate prescribing of antibiotics and adherence to guidelines improved However, this was not statistically significant unlike other areas such as polypharmacy and generic prescribing rates
Awad et al., Sudan, 2006 [279]	Twenty health centers in Khartoum State were randomly assigned to receive either (a) no intervention; (b) audit and feedback; (c) audit and feedback + seminar; or (d) audit and feedback + academic detailing The targeted interventions involving audit and feedback combined with academic detailing	Significant reduction in the mean number of physician encounters with an antibiotic prescribed by 6.3 and 7.7 (*p* < 0.001) at 1 and 3 months post-intervention respectively The mean number of encounters where antibiotics were inappropriately prescribed were also significantly reduced post-intervention (*p* < 0.001) However, reducing the number of interventions reduced their overall impact
Kafle et al., Nepal, 2009 [280]	Principally Education—Supervision/monitoring involving periodic visits by district supervisors to 41 primary healthcare (PHC) facilities The intervention included small-group training among prescribers followed by peer-group discussions alongside self-assessment of the data presented	In children under five, there was a significant improvement in use of antimicrobials in diarrhea There was also a significant improvement in the prescribing of antibiotics for URTIs without pneumonia
Yip et al., China, 2014 [281]	Principally Economics: In Ningxia Province, a randomized study was undertaken to evaluate the effects of capitation with pay-for-performance on antibiotic prescribing practices, health spending, outpatient visit volumes, and patient satisfaction	Approximately 15% reduction in antibiotic prescriptions Small reduction in total spending per visit
Boonyasiri et al., Thailand, 2014 [282]	Principally Education including: Training HCPs on the rational use of antibiotics Introduction of clinical practice guidelines Potential for throat swabs (stool cultures for acute diarrhea) Printed brochures for patients/relatives in waiting rooms containing likely etiology as well necessity and harm of antibiotics for URIs and acute diarrhea	The multifaceted program resulted in: Limited prescribing of antibiotics for URIs (13.0%) and for acute diarrhea (19.1%) Clinical responses on day 3 after receiving care revealed more than 97% of the patients who received antibiotics/those who did not receive antibiotics were cured or improved
Wei et al., China, 2019 [273]	Principally Education The multiple interventions comprised: clinical guidelines, monthly prescribing review meetings, doctor–patient communication skills training, and education materials for caregivers	This multifaceted approach appreciably reduced prescribing rates for antibiotics (ABR) in children with URTIs: A 49% reduction in ABR after 6 months in the intervention arm having adjusted for patient and prescribing doctor covariates The reductions persisted after 18 months but at a lower rate (−36%) Factors sustaining the reduction included physicians’ improved knowledge and communication skills combined with focused prescription review meetings
Tay et al., Malaysia, 2019 [283]	Principally Education—Educational toolkits included a training module for HCPs on URI and acute diarrhoea involving: One-hour educational sessions covering diagnostic criteria and treatment decision pathways Educational posters in Malay and English in the waiting area and consultation rooms as well as multimedia educational videos in the waiting areas Physician reminders	Appreciable reduction in antibiotic prescribing: URIs down from 29.1% to 13.7% Acute diarrhea down from 11.2% to 6.7%

**Table 4:** Examples of initiatives to reduce self-purchasing of antibiotics in pharmacies predominantly in LMICs (adapted from [64,100,183]).

Country and Year	Activity
Brazil and Mexico, 2013—private pharmacies [307]	Enforcement—assessing the impact of legislation to ban self-purchasing of antibiotics on dispensing patterns between 2007 and 2012 in private pharmacies in Brazil and Mexico (has always been the case among public pharmacies in Brazil [137,308]) Variable results seen Total antibiotic usage increased in Brazil (from 5.7 to 8.5 DDD/TID) but decreased in Mexico (10.5 to 7.5 DDD/TID) A change in the level of dispensing of −1.35 DDD/TID (*p* < 0.01) for Brazil and −1.17 DDD/TID (*p* < 0.00) for Mexico In Brazil, there was a decrease in the level of consumption of the penicillins, sulfonamides and macrolides by 0.64 DDD/TID (*p* = 0.02), 0.41 DDD/TID (*p* = 0.02) and 0.47 DDD/TID (*p* = 0.01) respectively The authors concluded that whilst the effect of the restrictions was similar across the countries; in Brazil, the trend of increased dispensing of antibiotics without a prescription was tempered after the restrictions were introduced whilst in Mexico the trend of decreasing dispensing accelerated
Brazil—Both private and public pharmacies—2015 to 2017	Enforcement: Moura et al. (2015) [308] showed a decrease in antibiotic use of 1.87 DDD/TID (*p* < 0.001) immediately after restrictions banning the sales of antibiotics without a prescription among private pharmacies (2008 to 2012), with a greater decrease in the more developed regions as well as in the State Capitals Not surprisingly, Moura et al. (2015) [308] found no difference in sales among public pharmacies where there had always been restrictions and it is generally impossible to sell antibiotics without a prescription (*p* = 0.643) Lopes-Junior et al. (2015) [309] found that sales of amoxicillin (commonly sold antibiotic) in private pharmacies fell by approximately 30% post-legislation despite a general growth in the pharmaceutical market, with falls in sales of other popular antibiotics including tetracyclines (30.5% decrease), sulfonamides (28.5% decrease), and macrolides (25% decrease) Mattos et al. (2017) [310] documented an increase in antibiotic sales among private pharmacies from 2008 to 2011 including the cephalosporins, quinolones, and aminopenicillins. Following changes in the law with restrictions in private pharmacies, there was a decrease in dispensing in 2012 for the cephalosporins (−19.4%), quinolones (−12.7%) and aminopenicillins (−11.1%) The differences in the findings in Brazil between the different studies may well reflect differences in datasets and methodologies
Thailand, 2015 [311]	Principally Education involving a multidisciplinary intervention among grocery stores in a rural province in Thailand using trained community leaders Grocery stores in the intervention group had 87% fewer antibiotics available postintervention compared with preintervention Grocery stores in the control group saw only an 8% reduction in antibiotic availability between the 2 time periods
Republic of Srpska, 2017 [157,312]	Education and Enforcement including guidelines for pharmacists and greater enforcement of the regulations resulted in self-purchasing of antibiotics for self-diagnosed URTIs significantly decreasing from 58% of requests to 18.5% Encouragingly, the most common reason for not dispensing an antibiotic was that antibiotics cannot be dispensed without a prescription
Kenya—2018 [99] and 2021 [313]	Monitoring of antibiotics dispensed among pharmacies linked to the University of Nairobi showed a low level of dispensing of antibiotics without a prescription, with 94.1% of antibiotics dispensed with a valid prescription (Education) No antibiotics were dispensed for patients with ARIs including influenza or a common cold, with over-the-counter (OTC) medicines such as cough and cold syrups and lozenges typically dispensed A more recent study conducted during the COVID-19 pandemic showed no dispensing of either antimalarials or antibiotics without a prescription
Saudi Arabia, 2020 [183]	Principally Economics and Enforcement In May 2018, the law and regulations surrounding self-purchasing without a prescription were enforced alongside fines Before enforcement, 70.7% of pharmacies reported self-purchasing was common, with 96.6% and 87.7% of participating pharmacies dispensed antibiotics without a prescription for pharyngitis and urinary tract infections (UTIs) respectively Following law enforcement and fines, only 12.9% reported self-purchasing still common, with only 12.1% and 5.2% dispensing antibiotics without prescriptions for pharyngitis and UTIs respectively When antibiotics were dispensed without a prescription, typically this only happened following considerable pressure from patients
India, Malaysia and Vietnam, 2021 [153,299]	There have been ongoing educational and other initiatives in recent years in India, Malaysia and Vietnam to try and reduce unnecessary self-purchasing of antimicrobials These initiatives seem to be working with no change or a decrease in the dispensing of antimicrobials among 83.3% to 100% of pharmacies surveyed in Malaysia and Vietnam in the initial months following the start of the COVID-19 pandemic despite the hype and concerns generally with increasing use of antibiotics In India—no change in up to 57.7% of pharmacists surveyed

**Table 5:** Positive impact of vaccine uptake and concerns with vaccine hesitancy.

Disease Area	Impact
Hib conjugate vaccine and pneumococcal polysaccharide vaccine	The introduction of the Hib conjugate vaccine resulted in an appreciable reduction in cases as well as a significant decrease in beta-lactamase-positive strains [10,351,352] A similar situation was seen following the introduction of the pneumococcal polysaccharide vaccine across countries [10,32,353,354,355]
*S. pneumoniae* and rotavirus	A pooled analysis illustrated the benefits of vaccination against *S. pneumoniae* and rotavirus in reducing antibiotic consumption, providing 19.7% and 11.4% protection against the use of antibiotics to treat acute respiratory infection and diarrhea respectively [356] Overall, it was estimated that these vaccines prevented 23.8 million antibiotic-treated ARI episodes annually among children aged 24–59 months, while the rotavirus vaccines prevented an estimated 13.6 million episodes of antibiotic-treated diarrhea amongst children 0–23 months [356].
COVID-19	Abbas et al. (2020) calculated that for every one excess death in Africa due to COVID-19, carrying on with routine vaccination could have prevented 84 deaths in children due to diphtheria, pertussis, hepatitis B, *Haemophilus influenzae* type b, measles, meningitis, *Streptococcus pneumoniae*, rotavirus, tetanus, rubella, and yellow fever [37] The unintended consequences of lockdown and other activities including reduced vaccine uptake must be factored into future decision making regarding potential strategies to address pandemics This includes a greater role for community pharmacists with vaccination programs during pandemics [36,357]

**Table 6:** Suggested strategies for LMICs to improve appropriate utilization of antimicrobials across sectors.

Time Scale	Potential Strategies
Short term	***Health authority/Government—the following (if not already done so):*** Governments and health authorities must be committed to reducing inappropriate use of antibiotics across sectors, building on current NAPs and other activities. This will necessarily involve resources (financial, technical and personnel) being made available for implementation and monitoring activities surrounding NAPs. This includes regulations to enhance ASP activities in hospitals and the community incorporating PPS surveys and other activities. In addition, recognizing that any ASPs may need to be culture and country specific, and sustainable in the long term, as well as multidisciplinary given concerns with their introduction in some LMICs [213,382] Ensure functional supply chains to meet the needs of prescribers including proactively addressing issues of shortage and building on activities across countries [262,383,384] Ascertain through PPS and other studies current rates of prescribing and dispensing of antimicrobials across sectors including ARIs in the community. This can include instigating PPS studies in hospitals to identify key areas for quality improvement. Similarly (if needed) assess current vaccination rates for critical vaccines In ambulatory care—ascertain current knowledge among key stakeholder groups including physicians, pharmacists, nurses and patients regarding AMR and the appropriateness of antibiotics especially for ARIs/COVID-19—using both quantitative and qualitative approaches. Subsequently instigate pertinent educational activities starting in universities as well as through patient organizations. This includes current training regarding antibiotics, AMR and ASPs in universities Assess the potential for introducing point-of-care testing and other clinical support systems especially following the current pandemic, including issues of affordability, as well as developing/supporting the implementation of a national integrated AMR surveillance system building on current activities monitoring rates of COVID-19 infections [49,153,299,385] Develop/update/disseminate robust guidelines surrounding the management of key infectious diseases across sectors, recognizing that active communication and dissemination of guidelines, as well as trust in the guidelines from all key stakeholder groups and ease of use, are key to enhancing adherence rates [170,386,387,388,389]. Consequently, these must be evidence based and with awareness of the WHO AWaRE list [173,390]. Subsequently, monitor prescribing against current guidelines and NAPs, enhanced by auditing, academic detailing and implementing electronic prescribing systems [386,389] This includes developing/refining pertinent quality indicators for use across sectors building on those already used in hospitals (Box 1) and developing new ones for ambulatory care in LMICs. In addition, evaluating potential approaches to improve adherence to any developed quality indicators including benchmarking, helped again by electronic prescribing systems [386,389], being part of CPD programs and possibly including financial incentives (small in relation to physician income) Work with key stakeholder groups including patient organizations to enhance adherence to treatments for conditions including HIV, malaria and TB given concerns with adherence to prescribed treatments and the subsequent impact on resistance rates. This could involve re-looking at issues such as increasing the number of clinics in rural areas as well as helping with transportation costs Work with patient organizations and others to also reduce misinformation regarding pandemics and their treatment as well as generally regarding vaccines given current concerns. As part of this, instigate country-wide vaccination programs including those for COVID-19, pneumococcal and influenza vaccines, with vaccines provided free of charge and available across sectors, which may require donor help initially Work with national/international pharmacy organizations to develop a range of additional activities that could be introduced where pertinent to reduce inappropriate self-purchasing of antimicrobials (building on Table 4). This could include introducing remuneration schemes for pharmacists if reducing dispensing of antibiotics appreciably impacts on their profit levels as well as IT and other systems to monitor the supply chain ***Physicians and nurse practitioners*** Ascertain current knowledge and beliefs about antibiotics, ASPs and AMR across sectors especially where there are concerns with the current situation and gaps in knowledge Work with key Governments and other organizations to develop (where pertinent) and make sure current national guidelines are evidence based and regularly updated as well as readily accessible through decision support systems and other mechanisms, as well as easy to use (as this is not always the case—[292]). In addition, encourage physicians through auditing and other practices to regularly consult national guidelines about optimal treatment approaches for their patients where there are concerns as this is also not always the case [181] Help with developing and instigating additional educational activities where pertinent [391]. This can include skills for communication with patients to help address current concerns Encourage physicians and nurse practitioners to become actively involved in ASPs as part of NAPs and wider programs Ascertain attitudes to the instigation of proven and cost-effective point-of-care testing and vaccination programs and subsequently address concerns to improve their roll out and uptake across countries Work with other healthcare professionals and patient organizations to address concerns with adherence to medicines for infectious diseases such as HIV and TB including instigating appropriate interventions and monitoring their impact ***Pharmacists (hospital)*** Become actively involved with progressing ASPs in the hospital, building on activities in countries such as South Africa [217] This can include addressing concerns with a lack of understanding regarding ASPs/AMR within hospitals [214,215] and providing educational support regarding appropriate antimicrobials to prescribe—especially if the main educational input in hospitals is via pharmaceutical companies [378,379] Become involved with undertaking PPS studies in hospitals including potential targets for key areas such as antimicrobial use to prevent SSIs (Table 1 and Table 2). The development of an App and other approaches may help here [204]. As part of this, regularly feedback concerns with antimicrobial usage patterns in hospitals to key stakeholder groups and work with them on potential ways forward Help with programs to enhance adherence to treatments for HIV, malaria and TB and subsequently monitor their impact Actively work with others in the hospital to proactively address possible shortages including agreeing in advance potential therapeutic interchange recommendations [264] ***Pharmacists (Community)*** Universities and others to work with community pharmacists to ascertain current knowledge and beliefs about antibiotics and AMR, especially for patients with ARIs, as well as the need to develop specific guidelines for ARIs and other self-limiting conditions (Table 3) Work with patients to address misinformation regarding antibiotics, AMR and the current pandemic, as well as enhance the role of pharmacists to administer vaccines/provide point-of-care testing, especially if they are a trusted source. As such, build on their role in the pandemic as a source of information generally regarding social distancing activities and possible symptomatic relief [36,49,153] Instigate additional educational activities among pharmacy students regarding antibiotics and AMR, as well as instigate/enhance CPD activities among community pharmacies with the help of relevant pharmaceutical societies and the Government to address concerns with lack of knowledge Encourage pharmacists, rather than technicians, through CPD and other measures to be the principal personnel within community pharmacies reviewing antimicrobial prescriptions and evaluating patients regarding their need for antimicrobials, especially for ARIs/COVID-19 Pharmacists to work with health authorities to computerize and track medicine use, i.e., via computerized IT systems, as well as work with health authorities and others to help transition away from focusing on business interests, especially in urban areas, to professional healthcare provision—again building on their role in the pandemic Advocate the introduction and benchmarking of national standards and guidelines for Good Pharmacy Practice (GPP) in community pharmacy settings as part of CPD, where this does not exist Help with programs to enhance adherence to treatments for HIV, malaria and TB, and subsequently monitor their impact ***Patients and the public*** Patient organizations helping with health education and public health messaging programs in schools/and more widely regarding the appropriate use of antibiotics and AMR, as well as key issues surrounding COVID-19 including vaccines (as well as other vaccines to reduce AMR). This includes helping to address mis- and dis-information especially where this can impact on future morbidity, mortality and personal finances Improve education and the extent of culturally sensitive information regarding antibiotics, AMR, and COVID-19 including vaccines through advocacy programs, patient information leaflets, social media, public health messaging and other community-based activities. As part of this, work with Governments and health authorities to ascertain the cost-effectiveness of campaigns directed at reducing inappropriate utilization of antibiotics for self-limiting conditions such as viral ARIs, as well as campaigns that can help reduce the level of misinformation especially surrounding prevention and treatments during pandemics Ascertain the rationale for any self-purchasing of antimicrobials, and use the findings to develop potential future strategies to reduce inappropriate requests for antibiotics including dispensing without a prescription. Public health messaging needs to be clear and via optimal channels depending on the country and circumstance Use educational initiatives to encourage parents/caregivers of young children to seek professional help for their ARIs/suspected COVID-19 including community pharmacists Work with key Government and other groups to enhance adherence to treatments for HIV, malaria and TB where challenges remain
Longer-term potential strategies	The findings from the situational analyses and ongoing educational activities can be used together with other research findings within each LMIC to develop pertinent long-term strategies for all key stakeholder groups. These include: Health authorities/Governments ○ Regularly monitoring antimicrobial utilization across sectors as part of any agreed NAP. This includes instigating electronic prescribing and tracking systems and addressing issues with telemedicine/vaccination programs where and when concerns are raised ○ Instigation of additional multiple strategies, where pertinent, to improve antibiotic utilization across sectors, including the provision of necessary resources required for implementing ASPs/IPC committees in hospitals, testing, clinical decision support systems, and regular updating of guidelines. This also includes developing quality indicators especially in ambulatory care given concerns with current WHO/International Network for Rational Use of Drugs (INRUD) criteria [167] ○ Regularly reviewing strategies to reduce inappropriate dispensing of antimicrobials in community pharmacies ○ Instigate additional programs and support to enhance adherence to treatments for HIV, malaria and TB and monitor their impact ○ Regularly review vaccination programs and update if necessary—this may mean the need for additional donor help ○ Increasing investment in research for new and existing antimicrobials, diagnostic tools, and vaccines Physicians and nurse practitioners ○ Regular reviews of current educational activities in medical/nursing schools regarding students’ knowledge of antibiotics, AMR, ASPs, COVID-19 and adapt/update where necessary ○ Regular monitoring of prescribing activities across sectors especially with regard to quality improvement programs in hospitals and ambulatory care. This can include increased accountability of prescribers with a requirement to justify their treatment approach alongside building on restrictions for certain antibiotics, building on the WHO Access, Watch, Reserve (AwaRe) antibiotic groupings [61,81,392,393] as well as on agreed quality indicators ○ Keep working with key stakeholders to enhance adherence to treatments such as those for HIV and TB Hospital Pharmacists ○ Work with key groups in hospitals to improve antimicrobial utilization through continued involvement in ASPs, IPCs and PPS studies—and monitoring of utilization patterns against agreed quality indicators ○ Concurrent with this, regularly review therapeutic interchange policies for possible antimicrobial shortages ○ Input into regular reviews of country NAPs and potential next steps ○ Work with pharmacy schools regarding possible additional educational activities to improve future utilization Community pharmacists ○ Regular reviews of current educational activities in pharmacy schools regarding student knowledge of antibiotics, AMR, ASPs, vaccines and COVID-19, and update where necessary ○ Act to ensure educational activities fully equip pharmacists for their extended roles as custodians of antimicrobials in ambulatory care. This can include instigating prescribing licenses for pharmacists to dispense agreed antimicrobials under guidance [123,124,394] ○ Help with additional activities including updating guidelines where relevant, especially when pandemics occur, as well as review initiatives including additional education and incentives to enhance appropriate dispensing of antimicrobials in the community ○ Development of pharmacist-led vaccination programs within the community, building on current concerns/issues with the COVID-19 pandemic ○ Work with key groups to strengthen the enforcement of pharmacy regulations and laws to curb irrational dispensing of antibiotics. Medicine tracking systems and mobile technologies can be effectively utilized to reduce inappropriate dispensing especially in rural areas where enforcement is problematic and maybe counter-productive Patients and the public ○ Regularly review the influence of any educational activities for their effectiveness, cost and value in all media and at all sites—especially important to reduce AMR and any misinformation surrounding COVID-19 ○ Continue to refine and update educational activities, including the work of patient organizations, and with all key stakeholder groups including Governments and healthcare professionals

## Data Availability

All sources of information have been referenced.

## Abbreviations

ABR	prescribing rates for antibiotics
AMR	Antimicrobial resistance
ARIs	acute respiratory infections
ASPs	antimicrobial stewardship programs
AST	Antimicrobial stewardship team
CDC	Centers for Disease Control and Prevention, US
CDDEP	Center for Disease Dynamics, Economics and Policy
CPD	Continuous professional development
CST	culture and sensitivity testing
DTCs	Drug and Therapeutic Committees
ECDC	European Centre for Disease Prevention and Control
4Es	Education, Engineering, Economics and Enforcement
GDP	Gross Domestic Product
GPP	Good Pharmacy Practice
HAIs	hospital-associated infections
HCPs	healthcare professionals
Hib	Haemophilus influenza type b
HIV	human immunodeficiency virus
ICU	intensive care unit
IDCPs	infectious diseases clinical pharmacists
IDCs	infectious diseases consultations
INRUD	International Network for Rational Use of Drugs
IPCs	Infection, Prevention and Control committees
IV	intravenous
LMICs	low- and middle-income countries
MDR	multidrug resistant
MDR-TB	multidrug-resistant TB
NAP	National Action Plans
NCDs	non-infectious communicable diseases
OECD	Organization for Economic Co-operation and Development
OTC	over the counter
PHCs	primary healthcare centres
PPSs	point prevalence surveys
SA	South Africa
SAP	surgical antimicrobial prophylaxis
SSIs	surgical site infections
TB	tuberculosis
UK	United Kingdom
URIs	upper respiratory infections
URTIs	upper respiratory tract infections
US	United States of America
UTIs	urinary tract infections
WHO	World Health Organization
XDR-TB	extensive drug-resistant TB
